# Cardio-respiratory outcomes associated with exposure to wildfire smoke are modified by measures of community health

**DOI:** 10.1186/1476-069X-11-71

**Published:** 2012-09-24

**Authors:** Ana G Rappold, Wayne E Cascio, Vasu J Kilaru, Susan L Stone, Lucas M Neas, Robert B Devlin, David Diaz-Sanchez

**Affiliations:** 1 Environmental Public Health Division, National Health and Environmental Effects Research Laboratory, United States Environmental Protection Agency, Research Triangle Park, North Carolina, USA; 2 National Exposure Research Laboratory, United States Environmental Protection Agency, Research Triangle Park, North Carolina, USA; 3 Office of Air Quality Planning and Standards, United States Environmental Protection Agency, Research Triangle Park, North Carolina, USA

**Keywords:** Disparities and susceptibility, Air pollution, Climate change, Asthma, Congestive heart failure, Wildfires

## Abstract

**Background:**

Characterizing factors which determine susceptibility to air pollution is an important step in understanding the distribution of risk in a population and is critical for setting appropriate policies. We evaluate general and specific measures of community health as modifiers of risk for asthma and congestive heart failure following an episode of acute exposure to wildfire smoke.

**Methods:**

A population-based study of emergency department visits and daily concentrations of fine particulate matter during a wildfire in North Carolina was performed. Determinants of community health defined by County Health Rankings were evaluated as modifiers of the relative risk. A total of 40 mostly rural counties were included in the study. These rankings measure factors influencing health: health behaviors, access and quality of clinical care, social and economic factors, and physical environment, as well as, the outcomes of health: premature mortality and morbidity. Pollutant concentrations were obtained from a mathematically modeled smoke forecasting system. Estimates of relative risk for emergency department visits were based on Poisson mixed effects regression models applied to daily visit counts.

**Results:**

For asthma, the strongest association was observed at lag day 0 with excess relative risk of 66%(28,117). For congestive heart failure the excess relative risk was 42%(5,93). The largest difference in risk was observed after stratifying on the basis of Socio-Economic Factors. Difference in risk between bottom and top ranked counties by Socio-Economic Factors was 85% and 124% for asthma and congestive heart failure respectively.

**Conclusions:**

The results indicate that Socio-Economic Factors should be considered as modifying risk factors in air pollution studies and be evaluated in the assessment of air pollution impacts.

## Background

Numerous studies have shown associations between air quality and cardio-respiratory morbidity and mortality. Particulate matter, particularly fine fraction (PM_2.5_), can aggravate asthma and has been linked to irregular heartbeats, heart attacks, and premature death. However, it is clear that not all communities are affected equally. In particular, communities with lower socio-economic status (SES) typically measured by income, education, and racial composition, have consistently been shown to be at increased risk from air pollutants [1-4] but other health factors associated with low SES such as limited access to clinical care or an unhealthy diet may also play an important role in determining a community’s health outcome to poor air quality [5-8]. Characterizing the relative importance of these health factors is an important step to understanding differences in community level risk and is critical to setting appropriate policy.

The most common difficulty encountered in evaluating community risk to air pollutants is that many health factors associated with poor health outcomes occur in communities where exposure to air pollutants is high. Several studies have shown that compared with those of higher SES, individuals in communities with low SES are more likely to be exposed to poorer air quality in ambient, residential and occupational environments [1,2]. Community risk studies are further complicated by the need to identify reliable health metrics that can be tracked consistently across communities [9]. Here, we sought to overcome these two obstacles and characterize community health factors indicative of acute health outcome risk by taking advantage of a natural phenomenon. We evaluated health responses following brief but acute wildfire smoke exposure in a region with low background pollution and utilized County Health Rankings (CHR) [10], based on a well established model of population health that characterize factors which determine community health. Concentrations of fine particulate matter (PM_2.5_) from smoke forecasting models averaged to the county are taken as the exposure matrix.

In 2008, burning deposits of peat during a wildfire in the Pocosin Lakes National Wildlife refuge in North Carolina produced smoke and haze intermittently for a number of weeks. Previously, we evaluated health effects that occurred during a three day episode in which the smoke plume moved inland and dispersed hazardous concentrations of air pollutants over the eastern and central part of the state [11]. In contrast to the current analysis the episode of exposure was determined using satellite measured aerosol optical density rather than PM_2.5_ concentrations. We found significant increases in emergency department (ED) visits for congestive heart failure (CHF), asthma, chronic obstructive pulmonary disease, pneumonia, and acute bronchitis in those counties which were most impacted by the wildfire. We hypothesized that the strong associations observed in the analysis may have been observed, in part, because the affected population was on average economically disadvantaged, rural, and with high prevalence of hypertension, diabetes, obesity, high blood pressure and other health conditions in comparison to the remainder of the state.

The use of 2010 County Health Rankings permits us to examine the modifying effect of health factors on ED visits for CHF and asthma at the community level. These rankings measure: health behaviors such as tobacco use, diet and exercise; access and quality of clinical care; social and economic factors such as education and income; and the physical environment. In addition, they provide a measure of the general health of the community by measuring two types of health outcomes (mortality, morbidity) at the county level. We focus on two clinical outcomes, CHF and asthma, that have distinct pathology but that have both been associated with susceptibility to the health effects of air pollution exposures [7,12,13]. We examine these indicators as modifiers of risk of adverse health outcomes following smoke exposure and show that the most important are socio-economic factors and measures of the overall health of counties.

## Methods

### Emergency department visits

Daily counts of ED visits were obtained from the NC Disease Event Tracking and Epidemiologic Collection Tool [14], a statewide, public health surveillance system. NCDETECT records daily ED visits from 111 of 114 civilian NC EDs with county of residence, gender, age, and discharge ICD-9-CM codes. In the study presented here we considered visits for two clinical outcomes: for asthma in patients over 18 years old (ICD-9-CM code 493); and for CHF patients over 44 years old (ICD-9-CM code 428). The study period was defined between the onset of the wildfire by lightening (June 1, 2008) and July 14th when the first rainfall, increased humidity, and controlled flooding contained the fire. During this period, average daily temperatures ranged from 69 to 86°F and no heat events were observed. More details are reported in [11]. The Human Subjects Institutional Review Board of the University of North Carolina at Chapel Hill, East Carolina University, and the Environmental Protection Agency approved the study.

### Exposure estimates

Concentrations of fine particulate matter dispersed from the Pocosin Lakes National Wildlife Refuge wildfire were obtained from the National Oceanic and Atmospheric Administration Smoke Forecasting System [15]. These estimates are based on smoke dispersion simulations from the Hybrid Single Particle Lagrangian Integrated Trajectory Model (HYSPLIT). The HYSPLIT model relies on satellite information of the wildfire location, U.S. Forest Service estimates for wildfire smoke emissions, and meteorological inputs from the North American Mesoscale mode. These are used as inputs to resolve vertical column integrated average concentrations hourly, at 0.15° latitude and longitude grid (~13.5 km). The estimated concentrations for the lowest 100 m surface layer were used and averaged over a 24 h period starting with midnight EST. We were unable to obtain a valid HYSPLIT simulation for June 4^th^ GMT, the first day when the fire became an open flame wildfire, underestimating concentrations on June 4^th^ and the night of June 3^rd^. Daily averages at the county level were subsequently calculated by averaging the 24 h period over the county boundaries using Monte Carlo approximation. Daily concentrations were obtained for the duration of the study period June 1^st^ – July 14^th^ 2008.

### Effect modifiers

We used the 2010 County Health Rankings for North Carolina to characterize community health factors that could potentially influence health outcomes [10]. These were developed by the Robert Wood Johnson Foundation and the University of Wisconsin Population Health Institute. CHR groups determinants of community health into four types of Health Factors: Health Behaviors, factors measuring access and quality to Clinical Care, Socio-Economic Factors, and the Physical Environment. In addition to these factors, two types of health outcomes (mortality and morbidity) are used. These measure how long people live (mortality) and how healthy people feel while alive (morbidity) and are general indicators of community health. Health Factors and Health Outcomes thus measure two distinct aspects, determinants vs outcomes, of county health. The County Health Rankings use data from variety of national data sources including Behavioral Risk Factor Surveillance System survey data (BRFSS) of Centers for Disease Control and Prevention, American Community Survey, as well as Dartmouth Atlas of Healthcare.

Health Factors, Outcomes, and individual measures along with their relative weights are listed in Table 1. Among the counties of eastern North Carolina, Mortality and Morbidity Outcome Rankings and Socioeconomic Factor rankings are strongly inter-correlated and mildly correlated with Health Behavior Factors (Additional file 1). The remaining factors, Clinical Care and Physical Environment Factors, are only weakly correlated among themselves and with all other factors. We classified 40 counties into “top” and “bottom” ranked groups relative to the median rank of each outcome and factor. Top ranking by all measures is a more desired outcome, indicating communities with better health ranking.

**Table 1:** **Health Ranking Weights for the 2010 County Health Rankings (source**http://www.countyhealthrankings.org**accessed July 2010)**

	**Cumulative**	**Measure**	**Weight**
	**Weight**		
Health Outcomes			
Mortality	50%	Years of potential life lost before age 75	50%
Morbidity	50%	Quality of life	50%
Health Factors			
Health Behavior	30%	Tobacco use	10%
		Diet and exercise	10%
		Alcohol us	5%
		Unsafe sex	5%
Clinical Care	20%	Access to care	10%
		Quality of care	10%
Socioeconomic Factors	40%	Education	10%
		Employment	10%
		Income	10%
		Family and social support	5%
		Community safety	5%
Physical Environment	10%	Environmental quality	5%
		Built environment	5%

### Statistical approach

The goal of the analysis was to consider the modifying effect of community level determinants of health on the risk for CHF and asthma visits relative to the concentrations of PM_2.5_. We applied a generalized linear mixed effects model with county specific intercept for Poisson count data to daily counts of ED visits (R version 2.11.1, lme4 package). Among the predictors, in the analysis we also included an indicator of daily concentration of PM_2.5_ above the common detection limit (0.1 μg/m^3^) to control for 0 inflated measurements arising from county-days without smoke. Log-transformed county population size estimates were used as the offset term in the analysis. A separate analysis was performed for two clinical outcomes by individual CHR outcome and factor. Relative risk of ED visits was examined with respect to the exposure concentrations on the day of the visit (lag 0), a day prior to the visit (lag 1), and the average of the two (average over lags 0 and 1) and compared with AIC/BIC criterions. In the case of asthma, exposure on the day of the visit was chosen as the best fitting model while in the case of CHF, the day prior to the visit was the main exposure variable. Results for health outcomes are summarized by excess relative risk or percent change ((RR-1)*100%) per 100 μg/m^3^ increase in daily concentration of PM_2.5_.

## Results

**Figure 1:**
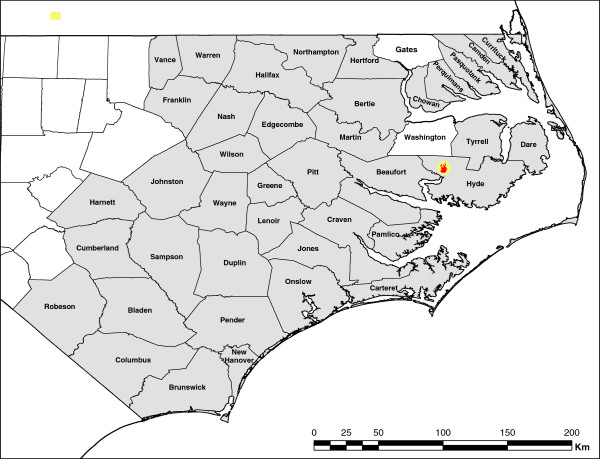
**An a real map of counties affected by the smoke. **Residents of two eastern counties, Washington and Gates, were excluded from the study; ED in Washington County did not participate in the surveillance program and Gates County was impacted by another fire.

**Figure 2:**
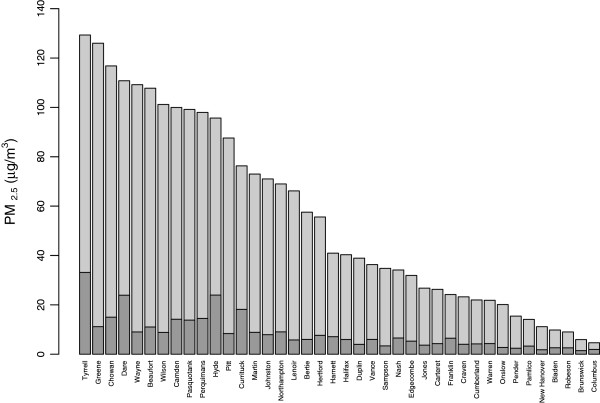
**Distribution of maximum daily concentrations (grey) and mean concentrations over the exposed (concentration > 1μg/m**^**3**^**) days.**

Counties experienced varying concentrations of smoke and length of time in the plume during the study period (Figure 1). Maximum daily smoke related PM_2.5_ concentration ranged from 4 to 129 μg/m^3^ (Figure 2). On average, during the study period, counties had 18 days of daily average concentrations above the detectable level and 3 days with average concentration higher than 20 μg/m^3^. Average daily concentrations and average concentrations over the study period were comparable between top and bottom grouped counties for most rankings. The exception was found for Mortality Outcome and the Clinical Care Factor rankings. Worse ranked counties by Mortality had significantly lower particle concentrations than their better ranked counterparts. Opposite was true for Clinical Care Factor, where worse ranked counties had significantly higher concentration of particles.

For asthma, the strongest association was observed on the day of the exposure (lag day 0) with 66 (28, 117)% increase in the rate of visits (per 100 μg/m3) while strongest association for CHF was observed with the day after the exposure (lag day 1) with 42 (5, 93)% increase in the rate of visits. The results for asthma are consistent with associations previously reported in the literature. The most common associations between exacerbations of asthma and wildfire smoke have been reported at lag days 0 and 1 and the average of the two [16-18]. Studies of wildfire smoke report more mixed associations with cardio-vascular effects. However, urban air pollution studies consistently show effects at lag day 0 and 1 following exposure. The lag structure in respiratory and particularly, cardiovascular outcomes, is likely determined not only by the time course of the physiological and biological health effect but cultural, social and environmental conditions that determine one’s use of the health care system.

**Figure 3:**
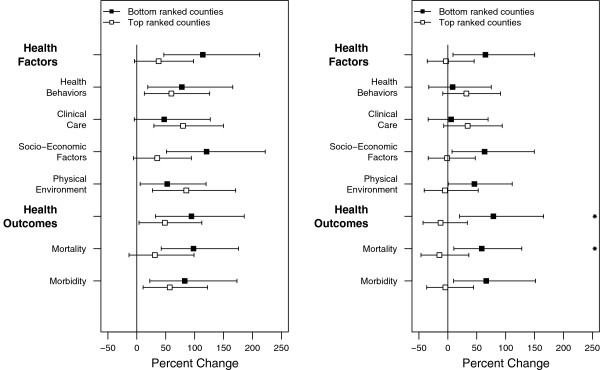
**Excess relative risk of emergency department visits for Asthma a) on the day of the exposure (at lag 0) and b) on the day following the exposure (lag 1). **Stars denote statistically significant difference in risk between two groups of counties. Estimates are reported per 100 μg/m^3^ of PM_2.5._

Relative Risk (RR) in asthma, associated with the day of exposure (lag 0), was highly elevated or statistically significant in ‘top’ and ‘bottom’ ranked groups of counties across all factors and outcomes (Figure 3A). The largest difference however, between bottom and top ranked counties among the individual factors and outcomes was observed for Socio-Economic Factors and Mortality Outcomes. Bottom ranked counties had 85% and 67% percent points higher risk than top ranked counties for these two measures respectively. Stratification by the aggregate measures, Health Factors and Health Outcomes, similarly showed sizeable differences in risk of 76% and 45% respectively. For all other measures, RR was comparable in magnitude between counties when similarly dichotomized.

**Figure 4:**
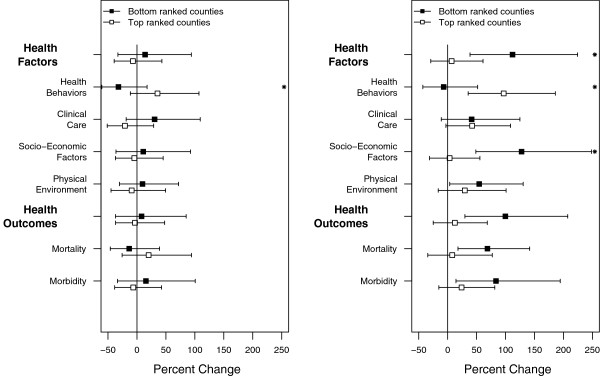
**Excess relative risk of emergency department visits for congestive heart failure a) on the day of the exposure (at lag 0), b) on the day following the exposure (lag1). **Stars denote statistically significant difference in risk between two groups of counties. Estimates are reported per 100 μg/m^3^ of PM_2.5._

We observed significantly increased risk in the counties ranked at the bottom by Socio-Economic Factors, Physical Environment Factors and both outcome measures, one day following the exposures (lag 1) (Figure 3B). By contrast, no changes were observed for the top ranked counties at this time. Difference in relative risk between top and bottom ranked counties by these two measures were 65% and 51% respectively. Stratification by both aggregate measures, Health Factors and Health Outcomes, similarly showed sizeable differences in risk of 68% and 92% respectively. Additionally, differences between top and bottom grouped counties were statistically significant when counties were stratified by Mortality and by Health Outcomes. No changes in RR were observed for either group of county as defined by Health Behaviors and Clinical Care Factors. We did not observe any changes in RR at lag day 0 for congestive heart failure (Figure 4A).

 However, associated with the day following the exposure (lag day 1), we observed significantly increased RR in bottom ranked counties by Socioeconomic Factors and Physical Environment, both outcome measures, as well as combined Health Factors and Health Outcomes (Figure 4B). In comparison, no changes in RR were observed for top ranked counties. Additionally, a statistically significant modifying effect was observed for counties grouped by Socio-Economic Factors, Health Behavior Factors and combined Health Factors.

The largest difference in risk between bottom and top ranked counties was again observed for Socio-Economic Factors with a difference of 124%. Mortality and Morbidity produced differences of 59% and 61% respectively. An opposite pattern was found for Health Behavior Factors where top ranked counties showed statistically significant risks while bottom ranked counties had no change.

Among the individual measures of Socio-Economic Factor, ‘Employment’, ‘Family and Social Support’, and ‘Community Safety’ showed consistent differences between top and bottom ranked counties for both asthma and CHF. The most pronounced difference in RR for asthma was observed for ‘Poverty’ measured by children below poverty where bottom ranked counties had 2 times higher relative risk than top ranked counties (2.68 vs. 1.38) at lag 0, and 53% higher at lag 1. In the case of CHF, the largest differences were observed for ‘income inequality’ where 223% higher risk was observed in bottom ranked counties.

## Discussion

The results presented here support the hypothesis that general and specific determinants of community health may be used as indicators of susceptibility to adverse health effects following environmental exposures. Numerous studies have shown evidence of association between particulate matter and cardio-respiratory morbidity and many have addressed the biological and genetic factors that influence the association. However, relevant social factors are less well understood. With a nearly complete record of ED visits and detailed daily maps of smoke related PM_2.5_ concentrations, we examined the impact of health factors on the risk of CHF and asthma in relation to the acute emissions of this pollutant. We demonstrate that among the different factors assessed, the strongest difference in relative risk for ED visits in both clinical outcomes was observed when counties were stratified on the basis of Socio-Economic Factors followed by indicators of community health Mortality and Morbidity Outcomes.

The category of Socio-Economic Factors measures ‘Employment’, ‘Community Safety’, ‘Income’, ‘Education’, and ‘Family and Social Support’. Of these, ‘Income’ was the best indicator of risk. Although different strategies have been used to quantify income and financial resources of the community, CHR uses poverty and income inequality as basic indicators of the community’s ability to meet the need for food, clothing, and shelter [10]. For asthma, poverty was the most important predictor while for CHF it was income inequality. Why these measures are better indicators than other factors more directly associated with clinical outcomes such as access to care or diet is unclear. There is a considerable literature on the detrimental health effects of poverty. A recent study [19], showed that poverty imposed the greatest burden of disease in the United States and is at least as important as smoking. Psychological and physical stress, highly present at conditions of severe poverty [20], and perceived inequalities by individuals have also been shown as important determinants of population health [21] explaining health inequalities at all social levels [22]. Stress impacts allostatic load in individuals, thus increasing the susceptibility to diseases. Populations with low SES share larger health burden as they have higher prevalence of chronic and under-treated medical conditions leading to an increased likelihood of adverse health effect in response to the exposures. Long-term exposure to psychological and social stress experienced in communities of low SES can also modify endocrine function and induce epigenetic changes transferable to the children [23-25]. In addition to biological mechanisms, poverty impacts many of the other factors measured by the CHR through the ability to pay for medical care, access to healthy foods, community safety and social support; all factors we show are associated with risk from PM_2.5_ exposure. The role of income inequality in defining health disparities has been long hypothesized and discussed area of research in social epidemiology. A traditional criticism has been the inability to separate individual and aggregate effects of income inequality in the society. However, more recent studies using multilevel data and multilevel statistical techniques suggest that evidence in support of association particularly at the aggregate levels such as counties and states[26].

The results for other Health Factors and specific measures were more complex. In particular, for asthma we observed an unexpected association between access to health care and ED visits on the day of the exposure. Those counties that ranked well in this category had higher rate of ED visits for asthma than the poorly ranked ones. Similarly, on the day following the exposure counties with higher primary care provider rate had increased risk of ED visits for CHF while poorly ranked counties did not. Access to health care is measured by the percentage of adults with no health insurance and the population per primary care provider in CHR. A possible explanation is that in the affected region, counties with a lower percentage of uninsured adults appear to have lower average income level and reflect communities comprised of many of the working poor. More specifically, using data from Census 2000 we have estimated a 1% (p value = 0.0063) increase in percent of uninsured adults for every $10 K increase in median household income at the county level. This is likely due to the large number of adults under the age of 65 that qualify for federal and state assistance and subsidized health plans in this region. While access and the quality of care may be important determinant of susceptibility at the individual level [8], at the community level we did not observe it to be a determinant of susceptibility.

A limitation of present study is in the ecological nature of the data on both exposures and effect modifiers, which are known only at the county level. For exposures, we have assumed that exposure to ambient air pollution are ubiquitous among the general population, that the mean personal exposures in a county are proportional to the county-wide concentration, and that personal exposures will be subject mainly to Berkson errors. Our analysis of HYSPLIT predictions against the satellite and monitoring data suggests that mis-specification of the magnitude of exposure as more likely source of error then mis-classification of day to day exposure status. For effect modifiers, the relationships at the county level examined in our study may not truly reflect relationships at the individual level due to an ecological bias. Our analysis shows that counties with higher poverty have stronger health associations with ambient smoke concentrations, suggestive that poor individuals may be more sensitive or vulnerable. However, an alternative explanation might be that all individuals in an impoverished county are equally sensitive regardless of individual level socioeconomic status, or even that wealthy individuals in impoverished counties are especially sensitive. Without individual level socioeconomic data on both cases and the referent population, one cannot distinguish between sensitivity or vulnerability conferred by individual or ecologic characteristics.

 Environmental exposures often fall disproportionately on economically disadvantaged populations and minorities [3]. However, recent air pollution studies indicate that even after accounting for differences in exposure, the health risks are not equally distributed among populations. For example, urban studies where exposure to traffic pollution is positively associated with socio-economic disparities [8,27] and those where negative association is observed [6], both report consistent results indicating enhanced health burden among socio-economically disadvantaged communities. The results from the presented study suggest that, following an acute exposure, the same results may be transferable to the populations that experience generally low levels of background pollution and are unlikely to be due to levels or longevity of the exposure alone. This suggests that SES increase the susceptibility to health outcomes independently of the vulnerability to exposures.

## Conclusions

In the work presented here we evaluate general and specific measures of community health as indicators of susceptibility to adverse events following air pollution exposures. The results suggest that, among various measures of health, Socio-Economic Factors played the most important role in defining susceptibility at the community level. These factors are not commonly considered as modifiers of risk in pollution studies because they can be confounded with number of other susceptibility enhancing factors. For example, SES is typically confounded with short and long term exposures as well as the prevalence of existing medical conditions. Here, we use a wildfire episode during which smoke blanketed the region irrespective of the community health characteristics to evaluate differences in risk. The results, suggesting that SES characteristics should be considered as risk modifiers to the impacts of air pollution exposures, are important steps to understanding differences in community risk and for setting appropriate policies.

## Abbreviations

PM_2.5_: Particulate matter with and average aerodynamic diameter of less then 10 μm, fine particulate matter; CHF: Congestive heart failure; RR: Relative risk; SEF: Socio-economic factors; SES: Socio-economic status; CHR: County health ranking; ED: Emergency department; NC: North Carolina; NCDETECT: North Carolina disease event tracking and epidemiologic collection tool; ICD-9-CM: International classification of diseases, ninth revision, clinical modification; HYSPLIT: Hybrid single particle Lagrangian integrated trajectory model.

## Competing interests

Authors have no competing interests to declare.

## Authors’ contributions

AGR has contributed substantially to the concept, design, analysis, interpretation of results and writing of the manuscript, WEC has contributed substantially to the concept and interpretation of results, VJK has contributed substantially to data collection and writing of the manuscript, SLS has contributed substantially to data collection and writing of the manuscript, LMN has contributed substantially to the concept, interpretation of results and writing of the manuscript, RBD has contributed substantially to the concept, interpretation of results and writing of the manuscript, DD-S has contributed substantially to the concept, interpretation of results and writing of the manuscript. All authors read and approved the final manuscript.

## Disclaimer

The research described in this article has been reviewed by the National Health and Environmental Effects Research Laboratory, U.S. EPA, and approved for publication. The contents of this article should not be construed to represent Agency policy nor does mention of trade names or commercial products constitute endorsement or recommendation for use.The NC DETECT Data Oversight Committee does not take responsibility for the scientific validity or accuracy of methodology, results, statistical analyses, or conclusions presented.

## Supplementary Material

Additional file 1 **Table S1. Spearman Rank Correlation coefficient between Community Health Ranking indices over 40 counties. **Table S2. County Ranking Summaries for counties of eastern North Carolina and the remaining counties in the state. Click here for file
