# Supplementary material for: Cardio-respiratory outcomes associated with exposure to wildfire smoke are modified by measures of community health
Source: Environ Health. 2012 Sep 24;11:71. doi: 10.1186/1476-069X-11-71 (PMC3506568; doi:10.1186/1476-069X-11-71)
Supplement: Additional file 1 — Table S1. Spearman Rank Correlation coefficient between Community Health Ranking indices over 40 counties. Table S2. County Ranking Summaries for counties of eastern North Carolina and the remaining counties in the state. [file 1476-069X-11-71-S1.docx]

**Supplemental Material**

**Table S1.** Spearman Rank Correlation coefficient between Community Health Ranking indices over 40 counties.

|  | | Mortality | | Morbidity | | Health Factors | | Health Behaviors | | Clinical Care | | Socio-economic Factors | | Physical- Environment | |
| --- | --- | --- | --- | --- | --- | --- | --- | --- | --- | --- | --- | --- | --- | --- | --- |
| **Health Outcomes** | **0.95** | | **0.89** | | 0.86 | | 0.56 | | -0.10 | | 0.85 | | 0.15 | |  |
| Mortality |  | | 0.73 | | 0.80 | | 0.56 | | -0.19 | | 0.81 | | 0.13 | |  |
| Morbidity |  | |  | | 0.82 | | 0.53 | | 0.04 | | 0.77 | | 0.20 | |  |
| **Health Factors** |  | |  | |  | | **0.69** | | **0.09** | | **0.92** | | **0.12** | |  |
| Health Behaviors |  | |  | |  | |  | | -0.03 | | 0.52 | | 0.17 | |  |
| Clinical Care |  | |  | |  | |  | |  | | -0.22 | | 0.11 | |  |
| Socioeconomic Factors |  | |  | |  | |  | |  | |  | | -0.03 | |  |

**Table S2.** County Ranking Summaries for counties of eastern North Carolina and the remaining counties in the state.

|  | **Eastern Counties**  **Median (IQR)** | **Remaining Counties**  **Median (IQR)** |
| --- | --- | --- |
| **Health Outcomes** | 65.0 (35.75, 89.50) | 45.0 (18.25, 63.50) |
| Mortality | 68.0 (36.50, 84.25) | 41.5 (21.25, 63.75) |
| Morbidity | 58.5 (39.50, 88.50) | 40.5 (19.25, 67.50) |
| **Health Factors** | 68.5 (43.75, 84.75) | 35.0 (16.25, 59.75) |
| Health Behaviors | 68.5 (56.75, 82.50) | 34.5 (16.25, 54.75) |
| Clinical Care | 63.5 (34.75, 84.75) | 43.0 (17.25, 63.75) |
| Socioeconomic Factors | 71.0 (37.00, 84.50) | 41.0 (20.25, 59.75) |
| Physical Environment | 51.5 (23.75, 67.25) | 50.0 (27.75, 78.75) |

Rankings are reported as the percentiles of distribution among 100 NC counties. Hence, the least desired ranking of 100 implies 100^th^ and bottom percentile of the distribution.

Topographically and demographically the 40 eastern counties included in the study were distinctly different from the counties in the western part of the state.
